# Incidence and treatment costs of severe bacterial infections among people who inject heroin: A cohort study in South London, England

**DOI:** 10.1016/j.drugalcdep.2020.108057

**Published:** 2020-07-01

**Authors:** Dan. Lewer, Vivian D. Hope, Magdalena. Harris, Michael. Kelleher, Amelia. Jewell, Megan. Pritchard, John. Strang, Katherine I. Morley

**Affiliations:** a Department of Epidemiology and Public Health, University College London, 1-19 Torrington Place, London WC1E 7HB, UK; b National Addiction Centre, Institute of Psychiatry, Psychology & Neuroscience, King’s College London, 4 Windsor Walk, Camberwell, London SE5 8AF, UK; c South London and Maudsley NHS Foundation Trust, Monks Orchard Road Beckenham, London BR3 3BX, UK; d Public Health Institute, Liverpool John Moores University, 3rd Floor, Exchange Station, Tithebarn Street, Liverpool L2 2QP, UK; e Department of Public Health, Environments and Society, London School of Hygiene & Tropical Medicine, 15-17 Tavistock Place, Kings Cross, London WC1H 9SH, UK; f Centre for Epidemiology and Biostatistics, Melbourne School of Global and Population Health, Level 3, 207 Bouverie Street, The University of Melbourne Victoria 3010 Australia; g RAND Europe, Westbrook Centre, Milton Road, Cambridge CB4 1YG, UK

**Keywords:** Drug injection, Heroin, Opiates, Bacterial infections

## Abstract

**Background:**

People who inject drugs often get bacterial infections. Few longitudinal studies have reported the incidence and treatment costs of these infections.

**Methods:**

For a cohort of 2335 people who inject heroin entering treatment for drug dependence between 2006 and 2017 in London, England, we reported the rates of hospitalisation or death with primary causes of cutaneous abscess, cellulitis, phlebitis, septicaemia, osteomyelitis, septic arthritis, endocarditis, or necrotising fasciitis. We compared these rates to the general population. We also used NHS reference costs to calculate the cost of admissions.

**Results:**

During a median of 8.0 years of follow-up, 24 % of patients (570/2335) had a severe bacterial infection, most commonly presenting with cutaneous abscesses or cellulitis. Bacterial infections accounted for 13 % of all hospital admissions. The rate was 73 per 1000 person-years (95 % CI 69–77); 50 times the general population, and the rate remained high throughout follow-up. The rate of severe bacterial infections for women was 1.50 (95 % CI 1.32–1.69) times the rate for men. The mean cost per admission was £4980, and we estimate that the annual cost of hospital treatment for people who inject heroin in London is £4.5 million.

**Conclusions:**

People who inject heroin have extreme and long-term risk of severe bacterial infections.

## Highlights

• People who inject heroin have extremely high risk of severe bacterial infections.
• Women have higher risk than men.
• Among those starting treatment for heroin dependence, high risk persists for at least ten years.
• Annual hospital treatment costs for injecting-related bacterial infections in London are £4.5m.

## 1 Introduction

Bacterial infections are common among people who inject illicit drugs. Cutaneous abscesses, cellulitis and other localised infections are some of the most frequent reasons for medical care in this population. These infection can be serious, sometimes requiring hospital treatment or leading to complications such as invasive infections and amputations. Cross-sectional studies show that between 7 % and 37 % of people who inject drugs report a soft tissue infection in the last 6–12 months, and lifetime prevalence may be as high as 70 % (Coull et al., 2014; Larney et al., 2017). Invasive bacterial infections at sites such as the bones, joints, heart, and blood, are also more common among people who inject drugs than the general population, and have a high mortality risk (Frontera and Gradon, 2000; Peterson et al., 2014). Time-series data suggest that the number of hospital admissions for injecting-related bacterial infections is increasing in the US and the UK (Ciccarone et al., 2016; Lewer et al., 2017).

Several elements of a causal pathway have been established. These include colonisation of the skin; transferring bacteria onto drugs when they are transported (for example in the mouth); frequent breaking of the skin when injecting (and sometimes through other injuries) (Phillips et al., 2017); and longer-term damage to the skin, soft tissue and veins with acids and particulate matter in drug preparations, which increases vulnerability to infection (Harris et al., 2019; Hope et al., 2008; Murphy et al., 2001; Packer et al., 2019). Clusters of unusual infections such as anthrax and botulism have been observed among people who use illicit drugs (Trayner et al., 2018), but there is likely to be many more infections and deaths caused by common species such as streptococci and staphylococci from the patient’s own skin or mouth (Gordon, 2005). Cross-sectional studies have identified that women, people who inject subcutaneously, homeless people, and those who inject stimulants have raised prevalence (Hope et al., 2008, 2016; Murphy et al., 2001). These groups may inject more frequently or have poorer access to sterile and sharp injecting equipment.

However, relatively few studies have reported the incidence and costs of bacterial infections among people who inject drugs, particularly in comparison to the large number of studies of blood-borne viral infections in this population (Degenhardt et al., 2016). This may be because most existing studies into bacterial infection use cross-sectional self-report data. In contrast to this, we used longitudinal electronic health record data to estimate the rate and treatment costs for severe bacterial infections in a cohort of people who inject heroin in South London, England.

## 2 Materials and methods

### 2.1 Data source

We used data from the Clinical Records Interactive Search (CRIS) resource at the South London and Maudsley NHS Foundation Trust Biomedical Research Centre. This is a research repository of anonymised data derived from the electronic health record system of a mental healthcare provider in South London, England (Perera et al., 2016). The study population was 2335 patients aged 18–64 entering community-based substance use treatment between 1 January 2006 and 31 March 2017, with reported use of heroin and drug injection. Patients were linked using NHS number, date of birth, sex and postcode to inpatient hospital admissions data from the national Hospital Episode Statistics for England database, and to mortality data from the UK Office for National Statistics. Linkage was conducted by NHS Digital, a public sector statistical agency. The end of follow-up was the participant’s 65th birthday, death, or 31 March 2017. Some patients have long periods of engagement with the drug treatment service, while other only attend one appointment, but data linkage was available for all patients regardless of their engagement with the service. We treated admissions within two days of discharge after a previous admission as a single admission. We also accessed hospital admission data for all residents in the healthcare provider’s catchment area of the London Boroughs of Croydon, Lambeth, Lewisham and Southwark (the ‘comparison group’).

### 2.2 Outcome measures

We defined severe bacterial infections as hospital admission with a primary cause of cutaneous abscess (ICD-10 code L02), cellulitis (L03), phlebitis or thrombophlebitis (I80), sepsis or septicaemia (A40, A41), endocarditis (I30.1, I39, I33.0, 140.0, I41.0), septic arthritis or osteomyelitis (M86, M00, M463, M46.5), and necrotising fasciitis (M72.6). We also counted all-cause hospital admissions.

### 2.3 Participant characteristics

Data were derived from routinely collected information. Drugs used by patients and ‘route of administration’ (i.e. whether the patient reports injecting) were from the treatment service’s National Drug Treatment Monitoring System (NDTMS) data set. This is a standardised patient assessment conducted periodically by drug treatment services in England (Marsden et al., 2009; Public Health England, 2018a). We identified that patients injected heroin if this was recorded on any NDTMS record during follow-up. In most of these cases, heroin injection was recorded on the earliest record, but we also included patients where it was recorded later, since some patients do not initially disclose injection. Date of birth and sex were taken from the healthcare provider’s central patient database. For descriptive purposes, we reported: (a) drugs other than heroin were listed for at least 10 % of participants, (b) whether homelessness or unstable housing was listed in patient databases, and (c) whether serious mental illness, defined as a diagnosis or bipolar disorder or schizophrenia, was listed in patient databases.

### 2.4 Statistical analysis

To calculate an expected number of admissions, we first calculated admission rates in the comparison group from 2006 to 2016 (as the closest available match to the study cohort) by age group, sex, and type of infection. The denominators were the sum of mid-year population estimates (Office for National Statistics, 2017) in the service provider’s catchment boroughs, and the numerators were the numbers of hospital admissions. We applied these rates to the time-at-risk within each age and sex group in the study cohort, accounting for patients ageing during follow-up and not counting time while patients were admitted to hospital. The standardised admission ratio (SAR) was the observed admissions divided by the expected admissions (i.e. indirect standardisation). We also stratified these results by sex.

We estimated the cost of each hospital admission using the NHS 2014/15 national reference costs (Department of Health and Social Care, 2015), in which hospitals report spend according to diagnoses, clinical procedures and the duration of admissions. The cost of each admission is calculated using a combination of the diagnosis codes, procedure codes, and length of admission. To contextualise these costs, we estimated the annual cost of hospital treatment for bacterial infections among all people who inject drugs in London, by applying the admission rates in our cohort to an existing capture-recapture population estimate of 11,351 people who inject drugs in London in 2011/12 (Hay et al., 2014), and using the mean cost of treatment for each diagnosis from our cohort. We used a Monte-Carlo method to estimate statistical uncertainty around this estimate, with details of this method provided in Supplementary Information.

To compare the duration of hospital admission for patients who inject drugs and the general population, we drew a random sample of admissions from the comparison group, stratified by age group, sex, primary diagnosis, and year, at a ratio of 1:1.

Analysis was conducted using R version 3.5.3 (R Core Team, 2019).

## 3 Results

The cohort included 2335 patients with a total follow-up time of 16,242 years (median 8.0, range 0–11.2). The mean age at baseline was 36.3 years (sd 8.4) and 1727 (74 %) were male, which is similar to the profile of people entering opioid treatment nationally (Public Health England and Department of Health, 2017) (Table 1). 352 patients died (15 %) during follow-up, of whom <10 had an underlying cause of a bacterial infection.

**Table 1:** Cohort characteristics at baseline.

Variable	Level	Number (%)
Age at index	18−24	217 (9)
	25−34	842 (36)
	35−44	921 (39)
	45−54	314 (13)
	55−64	41 (2)
	Mean (sd)	36.3 (8.4)
Age during follow-up	Mean (sd)	39.9 (8.4)
Sex	Male	1727 (74)
	Female	608 (26)
Ethnicity	White	2065 (88)
	Black	119 (5)
	Mixed	66 (3)
	Asian	28 (1)
	Other	57 (2)
Other drugs	Crack cocaine	1950 (84)
	Alcohol	1198 (51)
	Cannabis	513 (22)
	Benzodiazepines	386 (17)
Unstable housing		1410 (60)
Severe mental health problems		366 (16)
Total		2335 (100)

Of an initial cohort of 2469 patients, 134 (5 %) were not linked to NHS hospital data and were excluded from analysis. Excluded patients did not differ in terms of sex (p = 0.49), but were slightly younger at baseline (mean age 34.2 vs. 36.4 years; p = 0.005).

### 3.1 Hospitalisation

**Fig. 1:**
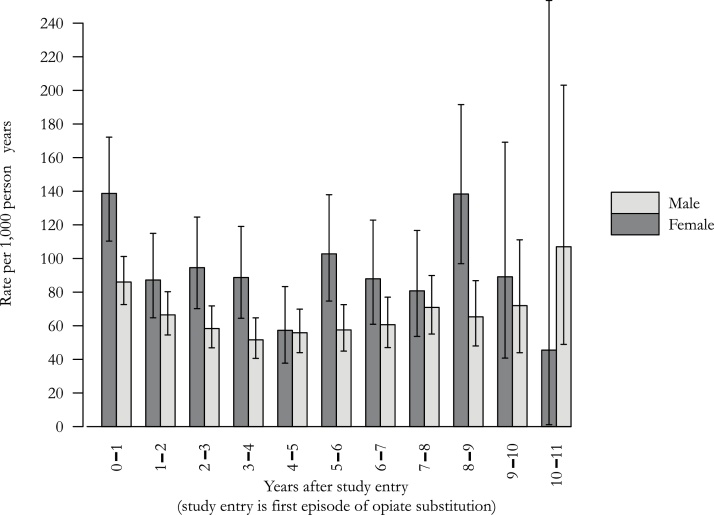
Rate of hospital admission for severe bacterial infection in a cohort of 2335 people who inject heroin in South London, England, by time after first treatment episode (error bars show 95% confidence intervals).

Patients were hospitalised 9315 times, of which 1180 (13 %) were primarily caused by a bacterial infection. The incidence density was 73 hospitalisations per 1000 person-years (95 % CI 69–77). The rate of bacterial infections was high throughout follow-up, with no evidence of a change in incidence over time for either men or women (Fig. 1).

Compared to the general population, the study cohort was 50.0 (95 % CI 47.2−52.9) times more likely to be admitted to hospital for treatment of a bacterial infection. Hospitalisation rates were substantially raised for each type of bacterial infection (Table 2). The rate of all-cause admission was also raised, but much less so, at 3.7 times the general population.

**Table 2:** Hospital admissions and costs of treatment for severe bacterial infections in a cohort of 2335 people who inject heroin in South London, England, with 16,434 years of follow-up.

Primary diagnosis	Observed admissions	Expected admissions	SAR (95 % CI)	Mean cost, £ (sd)	Median cost, £ (IQR)
Abscess	487	9.3	52.4 (47.8−57.2)	4307 (3035)	3898 (2,660−5,296)
Cellulitis	282	7.8	36.0 (31.9−40.4)	3579 (2503)	2731 (1,880−4,432)
Phlebitis and thrombophlebitis	233	2.0	115.2 (100.9−131.0)	3261 (2969)	2277 (1,772−4,136)
Septicaemia and bacteraemia	56	2.3	24.3 (18.3−31.5)	8687 (5060)	9250 (5,221−9,763)
Osteomyelitis and septic arthritis	42	0.2	174.4 (125.7−235.8)	14,134 (52,843)	5694 (3,980−7,129)
Endocarditis	82	1.9	43.7 (34.8−54.3)	12,963 (7765)	11,951 (6,893−15,197)
Necrotising Fasciitis	9	<0.1	599.4 (274.1−1,137.9)	10,815 (7159)	11,926 (4,274−14,839)
All bacterial infections*	1180	23.6	50.0 (47.2−52.9)	4980 (12,431)	3022 (2,148−5,296)
All-cause	9274	2467.5	3.8 (3.7−3.8)	**	**

SAR = Standardised admission ratio.

* The total number of bacterial infections is less than the sum of each individual diagnosis because some admissions have two primary diagnoses (resulting from the process of merging hospital admissions that were within two days of each other).

** We did not calculate the cost of admissions with primary diagnosis unrelated to bacterial infection.

**Fig. 2:**
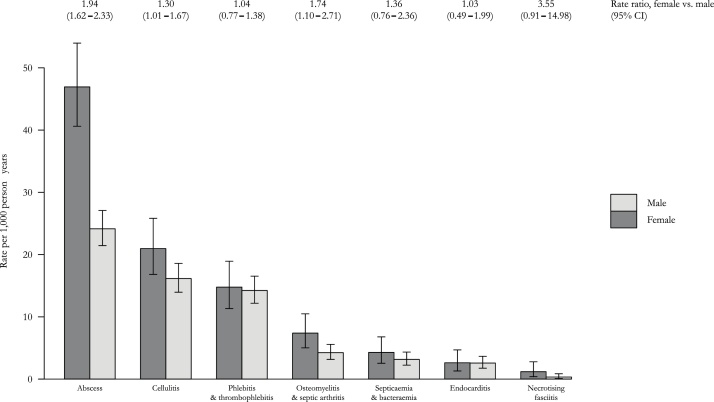
Rate of hospital admission for severe bacterial infection in a cohort of 2335 people who inject heroin, by primary diagnosis. (error bars show 95% confidence intervals).

The rate of hospitalisation among women was 1.50 (95 % CI 1.32–1.69) that of men, with similar or higher rates across diagnoses (Fig. 2). Although women also had a higher rate of all-cause hospital admission than men, this was in proportion to differences between women and men in the general population. All-cause SARs for men and women were therefore similar, at 3.7 and 4.0 respectively. In contrast, women who inject heroin had higher rates of admission for bacterial infections than men, and the differences were disproportionate to underlying differences between men and women in the general population. The SARs for bacterial infections were therefore higher for women than for men (see Supplementary Information).

Compared to hospital inpatients from the general population with the same age, sex, primary cause of hospital admission and year of admission, patients who inject heroin had a longer duration of admission (mean 7.4 days) and were more likely to self-discharge (13 % vs. 1 %). Comparisons of the duration, admission method and discharge methods for people who inject heroin and the general population are provided in Supplementary Information.

### 3.2 Cost of treatment

The mean cost per admission was £4980 (sd. £12,431), with higher costs for invasive infections. The cost per admission was heavily right-skewed (common in healthcare cost data) and means were higher than medians. Modelling suggested 869 admissions for treatment of bacterial infections per year among 11,351 people who inject drugs in London, with a total cost of £4.5 million (95 % CI £3.7-£5.4 million), based on 2014/15 prices.

## 4 Discussion

In this cohort of people who inject heroin in South London, bacterial infections were a major cause of morbidity but not mortality. Our results show a 50-fold increased risk of severe bacterial infections when compared to the general population, with high risk persisting for several years after starting treatment.

Hospital treatment of bacterial infections in people who inject drugs can be complex and expensive. Clinicians sometimes retain these patients in hospital for longer to ensure antibiotic courses are completed. Patients may leave hospital against medical advice if opiate substitution is unavailable (Summers et al., 2018) and often have poor continuing care, leading to readmission and antimicrobial-resistant infections. In our sample, 13 % of admissions ended in discharge against medical advice, compared to 1 % of admissions in the comparison group. Our results suggest that the cost of hospitalisations for these conditions is £4.5 million per year in London (2014/15 prices), which is substantial considering that the total expenditure on drug misuse treatment in London in 2017/18 was £64 million (Ministry of Housing, Communities and Local Government, 2018).A previous study estimated costs of hospital treatment for bacterial infection among people who inject drugs in England using the mean cost of treatment in the general population, at £944 to £1566 per admission (2004/05 prices) (Hope et al., 2008). Our data show substantially higher costs among people who inject drugs, at £4980 per admission (2014/15 prices), which is similar to a study of 128 episodes of bacterial infection in people who inject drugs at one London hospital (Marks et al., 2013).

The incidence rate of severe bacterial infections (73/1000 person-years) was higher than rates observed in cohorts of people who inject opiates in Sweden (24/1000) and Canada (61/1000) (Dahlman et al., 2018; Lloyd-Smith et al., 2010) and lower than a rate observed in Switzerland (86/1000) (Bassetti et al., 2002). The variation is likely due to differing demographics, injecting behaviours, types of heroin, local services, and the period when the study was conducted. The higher risk associated with female sex is consistent across these studies.

Many studies have shown that women form a minority among people who inject drugs, with higher risk of acquiring infections. As well as higher risk of bacterial infections, some studies have shown than women who inject drugs have higher risk of acquiring blood-borne viral infections than men, with the difference at least partly due to injecting-related risks such as sharing syringes, injection by partners, and younger age at first injection (Doherty et al., 2000; Tracy et al., 2014). Women who inject drugs may face greater self-stigma and social stigma relating to drug injection and injection-related injuries (Iversen et al., 2015), and may therefore avoid health services. Policy recommendations to reduce HIV risk among women who inject drugs have argued that the most successful interventions focus on contextual factors such as women’s intimate relationships, housing, employment, and childcare arrangements, rather injecting behaviours (Pinkham et al., 2012), and this may also be true for bacterial infections.

A large proportion of our sample (60 %) had experienced homelessness or housing problems. The association between housing problems and bacterial infections among people who inject drugs has been shown in previous studies (Dahlman et al., 2018; Hope et al., 2008). Sleeping in homeless shelters and other temporary accommodation may be associated with increased bacterial colonisation (Leibler et al., 2019). Homeless people are also more likely to inject in public places, which is associated with rushing the procedure, not cleaning skin before injecting, a lack of clean water for preparing drug solutions, and not having a clean surface to assemble the drugs (Small et al., 2007), which increase the risk of infection. Interventions to improve housing in this population may reduce the risk of bacterial infections, as well as improving many other health and social outcomes. Among people using accommodation with shared bathroom facilities, improved shower hygiene may decrease the risk of colonisation (Leibler et al., 2019).

Qualitative research has found multiple barriers to healthcare among people who inject drugs. People may delay treatment due to normalisation of pain, fear of stigma in services, and concern about inadequate opioid substitution and pain control when admitted to hospital (Neale et al., 2007; Summers et al., 2018). Hospitals sometimes employ drug and alcohol liaison workers a to improve accessibility for people who use drugs, and this may lead to more effective treatment (Reeve et al., 2016). Early treatment may also be encouraged by specialist community clinics that provide antibiotics and wound care. These services are sometimes commissioned as part of community drug services, but may have become less available in England as funding for addictions services has reduced (Advisory Council on the Misuse of Drugs, 2017).

In addition to interventions focusing on gender, housing, and health service accessibility, there are several effective interventions that reduce injecting-related risk. These include interventions that reduce the need for injecting, such as opiate substitution; and interventions that improve injecting safety and hygiene, such as safe injecting facilities and provision of sterile injecting equipment (Dunleavy et al., 2017).

We observed continued high risk of severe bacterial ten years after initiation of treatment. This reflects the long-term nature of heroin dependence and highlights the need for continued health assessments and inspection of injecting sites.

### 4.1 Strengths and limitation

Recruiting and retaining people who use drugs in traditional cohort studies can be challenging, and many studies are small and suffer from loss-to-follow-up. Strengths of our study include the large sample size, long follow-up, and complete data on hospitalisations and deaths. The linked hospital records were available from all NHS hospitals in England (rather than only local hospitals), which is important because people who use drugs are mobile and may use health services in other parts of the country. In our study, 55 % of admissions occurred outside of the four local London boroughs.

Our sample is drawn from a community drug treatment service, and therefore excludes people who have never sought treatment. In England, an estimated three-quarters of people who use illicit opiates have had at least one episode of treatment and half are currently engaged with treatment (Public Health England, 2018b). Those who have never engaged with treatment may include both higher risk patients who are not accessing harm reduction services, and lower risk patients who have lower need for services. Given the high proportion of the population who have used drug treatment services, our results are likely to be a reasonable estimate of the rate of infection among people who inject heroin in London.

We focused on severe infections that require inpatient treatment. Community surveys suggest that soft tissue infections are very common in people who inject drugs (Larney et al., 2017), and many self-treat (Monteiro et al., 2020) or wait for symptoms to resolve. Additionally, some patients will have sought treatment for less severe infections from general practitioners, but we did not have access to primary care data for this study. Consequently, this study only captures the most severe infections and provides a lower bound for healthcare utilisation.

Although our data included potential risk factors for bacterial infections, such as housing status and mental health problems, we did not seek to analyse their association with the risk of bacterial infections. Previous studies have reported risk factors for bacterial infections in this population, and our data lacked detailed information relating to injecting risk, such as duration and frequency of injecting. This study highlights that cohorts based on electronic health records such as ours have strengths when reporting of incidence and costs, but may be limited when analysing risk factors or ‘risk environments’, which requires a detailed understanding of participants’ context.

## 5 Conclusions

People who inject heroin have extreme and long-term risk of severe bacterial infections. Women are at higher risk than men.

## Data sharing

Researchers with appropriate registrations and permissions can access data from CRIS. Contact the NIHR Maudsley Biomedical Research Centre for further information. https://www.maudsleybrc.nihr.ac.uk/

## Ethical approval

The dataset was approved as an anonymised data set for secondary data analyses by the Oxfordshire Research Ethics Committee C (reference number: 08/H0606/71+5). This analysis was approved by the South London and Maudsley NHS Foundation Trust Biomedical Research Centre CRIS Oversight Committee (reference number: 17−073).

## Funding

DL and MH are funded by the 10.13039/501100012618NIHR [Doctoral Research Fellowship DRF-2018-11-ST2-016, CDF-2016-09-014]. This work was supported by a grant from the 10.13039/100010269Wellcome Trust [109823/Z/15/Z] to KIM. JS is supported by the NIHR Biomedical Research Centre for Mental Health at South London and Maudsley NHS Foundation Trust and King's College London; and is an NIHR Senior Investigator. The views expressed are those of the authors and not necessarily those of the NHS, the NIHR or the Department of Health and Social Care.

## Role of the funding source

The funders had no role in the study design, the collection, analysis and interpretation of data, in the writing of the report and in the decision to submit the article for publication. This manuscript represents independent research part funded by the National Institute for Health Research (NIHR) Biomedical Research Centre at South London and Maudsley NHS Foundation Trust and King's College London.

## Contribution statement

DL and KIM conceived the study and drafted the analysis plan. All authors revised and approved the analysis plan. AJ and MP created the analytical dataset. DL conducted data analysis and drafted the manuscript. All authors critically revised the manuscript. All authors have read and approved the final manuscript.

## Declaration of Competing Interest

MK is employed by the South London and Maudsley NHS Trust (SLaM) as clinical lead for Lambeth Addictions Consortium, which provides treatment for the patients included in this study. JS is a clinician and researcher and has worked extensively on clinical trials and wider research. JS’s employer (King’s College London) receives, unconnected to this specific study but connected to his wider work, project grant support and/or honoraria and/or consultancy payments from government agencies, charitable sources and also from pharmaceutical companies related to funding for clinical trials and research studies (for fuller information see www.kcl.ac.uk/ioppn/depts/addictions/people/hod.aspx).

## References

[bib0005] Advisory Council on the Misuse of Drugs (2017). Commissioning impact on drug treatment. The Extent to Which Commissioning Structures, the Financial Environment and Wider Changes to Health and Social Welfare Impact on Drug Misuse Treatment and Recovery.

[bib0010] Bassetti S., Hoffmann M., Bucher H.C., Fluckiger U., Battegay M. (2002). Infections requiring hospitalization of injection drug users who participated in an injection opiate maintenance program. Clin. Infect. Dis..

[bib0015] Ciccarone D., Unick G.J., Cohen J.K., Mars S.G., Rosenblum D. (2016). Nationwide increase in hospitalizations for heroin-related soft tissue infections: associations with structural market conditions. Drug Alcohol Depend..

[bib0020] Coull A.F., Atherton I., Taylor A., Watterson A.E. (2014). Prevalence of skin problems and leg ulceration in a sample of young injecting drug users. Harm Reduct. J..

[bib0025] Dahlman D., Berge J., Björkman P., Nilsson A.C., Håkansson A. (2018). Both localized and systemic bacterial infections are predicted by injection drug use: a prospective follow-up study in Swedish criminal justice clients. PLoS One.

[bib0030] Degenhardt L., Charlson F., Stanaway J., Larney S., Alexander L.T., Hickman M., Cowie B., Hall W.D., Strang J., Whiteford H., Vos T. (2016). Estimating the burden of disease attributable to injecting drug use as a risk factor for HIV, hepatitis C, and hepatitis B: findings from the Global Burden of Disease Study 2013. Lancet Infect. Dis..

[bib0035] Department of Health and Social Care (2015). NHS Reference Costs 2014 to 2015.

[bib0040] Doherty M.C., Garfein R.S., Monterroso E., Brown D., Vlahov D. (2000). Correlates of HIV infection among young adult short-term injection drug users. AIDS.

[bib0045] Dunleavy K., Munro A., Roy K., Hutchinson S., Palmateer N., Knox T., Goldberg D., Taylor A. (2017). Association between harm reduction intervention uptake and skin and soft tissue infections among people who inject drugs. Drug Alcohol Depend..

[bib0050] Frontera J.A., Gradon J.D. (2000). Right-side endocarditis in injection drug users: review of proposed mechanisms of pathogenesis. Clin. Infect. Dis..

[bib0055] Gordon R.J. (2005). Bacterial infections in drug users. N. Engl. J. Med..

[bib0060] Harris M., Scott J., Wright T., Brathwaite R., Ciccarone D., Hope V. (2019). Injecting-related health harms and overuse of acidifiers among people who inject heroin and crack cocaine in London: a mixed-methods study. Harm Reduct. J..

[bib0065] Hay G., Rael dos Santos A., Worsley J. (2014). Estimates of the Prevalence of Opiate Use and/or Crack Cocaine Use.

[bib0070] Hope V., Kimber J., Vickerman P., Hickman M., Ncube F. (2008). Frequency, factors and costs associated with injection site infections: findings from a national multi-site survey of injecting drug users in England. BMC Infect. Dis..

[bib0075] Hope V.D., Parry J.V., Ncube F., Hickman M. (2016). Not in the vein: ‘missed hits’, subcutaneous and intramuscular injections and associated harms among people who inject psychoactive drugs in Bristol, United Kingdom. Int. J. Drug Policy.

[bib0080] Iversen J., Page K., Madden A., Maher L. (2015). HIV, HCV, and health-related harms among women who inject drugs: implications for prevention and treatment. JAIDS J. Acquir. Immune Defic. Syndr..

[bib0085] Larney S., Peacock A., Mathers B.M., Hickman M., Degenhardt L. (2017). A systematic review of injecting-related injury and disease among people who inject drugs. Drug Alcohol Depend..

[bib0090] Leibler J.H., Liebschutz J.M., Keosaian J., Stewart C., Monteiro J., Woodruff A., Stein M.D. (2019). Homelessness, personal hygiene, and MRSA nasal colonization among persons who inject drugs. J. Urban Health.

[bib0095] Lewer D., Harris M., Hope V. (2017). Opiate injection–associated skin, soft tissue, and vascular infections, England, UK, 1997–2016. Emerg. Infect. Dis..

[bib0100] Lloyd-Smith E., Wood E., Zhang R., Tyndall M.W., Sheps S., Montaner J.S., Kerr T. (2010). Determinants of hospitalization for a cutaneous injection-related infection among injection drug users: a cohort study. BMC Public Health.

[bib0105] Marks M., Pollock E., Armstrong M., Morris-Jones S., Kidd M., Gothard P., Noursadeghi M., Doherty J.F. (2013). Needles and the damage done: reasons for admission and financial costs associated with injecting drug use in a Central London Teaching Hospital. J. Infect..

[bib0110] Marsden J., Eastwood B., Bradbury C., Dale-Perera A., Farrell M., Hammond P., Knight J., Randhawa K., Wright C. (2009). Effectiveness of community treatments for heroin and crack cocaine addiction in England: a prospective, in-treatment cohort study. Lancet.

[bib0115] Ministry of Housing, Communities & Local Government (2018). Revenue Outturn Social Care and Public Health Services (RO3) 2017 to 2018.

[bib0120] Monteiro J., Phillips K.T., Herman D.S., Stewart C., Keosaian J., Anderson B.J., Stein M.D. (2020). Self-treatment of skin infections by people who inject drugs. Drug Alcohol Depend..

[bib0125] Murphy E.L., DeVita D., Liu H., Vittinghoff E., Leung P., Ciccarone D.H., Edlin B.R. (2001). Risk factors for skin and soft-tissue abscesses among injection drug users: a case-control study. Clin. Infect. Dis..

[bib0130] Neale J., Tompkins C., Sheard L. (2007). Barriers to accessing generic health and social care services: a qualitative study of injecting drug users: drug injectors and barriers to service use. Health Soc. Care Community.

[bib0135] Office for National Statistics (2017). Population Estimates: Analysis Tool.

[bib0140] Packer S., Pichon B., Thompson S., Neale J., Njoroge J., Kwiatkowska R.M., Oliver I., Telfer M., Doumith M., Buunaaisie C., Heinsbroek E., Hopewell-Kelly N., Desai M., Hope V., Williams O.M., Kearns A., Hickman M., Gobin M. (2019). Clonal expansion of community-associated meticillin-resistant Staphylococcus aureus (MRSA) in people who inject drugs (PWID): prevalence, risk factors and molecular epidemiology, Bristol, United Kingdom, 2012 to 2017. Eurosurveillance.

[bib0145] Perera G., Broadbent M., Callard F., Chang C.-K., Downs J., Dutta R., Fernandes A., Hayes R.D., Henderson M., Jackson R., Jewell A., Kadra G., Little R., Pritchard M., Shetty H., Tulloch A., Stewart R. (2016). Cohort profile of the South London and maudsley NHS Foundation Trust Biomedical Research Centre (SLaM BRC) case register: current status and recent enhancement of an electronic mental health record-derived data resource. BMJ Open.

[bib0150] Peterson T.C., Pearson C., Zekaj M., Hudson I., Fakhouri G., Vaidya R. (2014). Septic arthritis in intravenous drug abusers: a historical comparison of habits and pathogens. J. Emerg. Med..

[bib0155] Phillips K.T., Anderson B.J., Herman D.S., Liebschutz J.M., Stein M.D. (2017). Risk factors associated with skin and soft tissue infections among hospitalized people who inject drugs. J. Addict. Med..

[bib0160] Pinkham S., Stoicescu C., Myers B. (2012). Developing effective health interventions for women who inject drugs: key areas and recommendations for program development and policy. Adv. Prev. Med..

[bib0165] Public Health England (2018). Drug and Alcohol Treatment Outcomes: Measuring Effectiveness.

[bib0170] Public Health England (2018). Unlinked anonymous HIV and viral hepatitis monitoring among PWID: 2018 report. Health Prot. Rep..

[bib0175] Public Health England, Department of Health (2017). Adult Substance Misuse Statistics from the National Drug Treatment Monitoring System (NDTMS).

[bib0180] R Core Team (2019). R: A Language and Environment for Statistical Computing.

[bib0185] Reeve R., Arora S., Butler K., Viney R., Burns L., Goodall S., van Gool K. (2016). Evaluating the impact of hospital based drug and alcohol consultation liaison services. J. Subst. Abuse Treat..

[bib0190] Small W., Rhodes T., Wood E., Kerr T. (2007). Public injection settings in Vancouver: physical environment, social context and risk. Int. J. Drug Policy.

[bib0195] Summers P.J., Hellman J.L., MacLean M.R., Rees V.W., Wilkes M.S. (2018). Negative experiences of pain and withdrawal create barriers to abscess care for people who inject heroin. A mixed methods analysis. Drug Alcohol Depend..

[bib0200] Tracy D., Hahn J.A., Fuller Lewis C., Evans J., Briceño A., Morris M.D., Lum P.J., Page K. (2014). Higher risk of incident hepatitis C virus among young women who inject drugs compared with young men in association with sexual relationships: a prospective analysis from the UFO Study cohort. BMJ Open.

[bib0205] Trayner K.M.A., Weir A., McAuley A., Godbole G., Amar C., Grant K., Penrice G., Roy K. (2018). A pragmatic harm reduction approach to manage a large outbreak of wound botulism in people who inject drugs, Scotland 2015. Harm Reduct. J..

