# Supplementary material for: Incidence and treatment costs of severe bacterial infections among people who inject heroin: A cohort study in South London, England
Source: Drug Alcohol Depend. 2020 Jul 1;212:108057. doi: 10.1016/j.drugalcdep.2020.108057 (PMC7301433; doi:10.1016/j.drugalcdep.2020.108057)
Supplement: Supplementary file 1 [file mmc1.pdf]

Supplementary information

1. Standardised admission ratios stratified by sex
2. Duration of hospital admission
3. Modelled annual costs for London

1 Standardised admission ratios stratified by sex

Table: hospital admissions for severe bacterial infections in a cohort of 2335 people who inject drugs, stratified by sex

Sex	Primary diagnosis	Observed admissions	Rate per 1,000 person-years	Expected admissions	SAR
Male	Cutaneous abscess	290	24.1	7.3	39.5 (35.1-44.3)
	Cellulitis	194	16.1	6.7	29.0 (25.1-33.4)
	Phlebitis and thrombophlebitis	171	14.2	1.6	106.2 (90.9-123.4)
	Septicaemia and bacteraemia	38	3.2	1.7	22.1 (15.7-30.4)
	Osteomyelitis and septic arthritis	31	2.6	0.2	155.5 (105.6-220.7)
	Endocarditis	51	4.2	1.7	30.4 (22.6-40.0)
	Necrotising Fasciitis	**	**	**	345.1 (94.0-883.6)
	All bacterial infections*	775	64.4	19.2	40.3 (37.5-43.2)
	All-cause	6,095	506.4	1,573.6	3.9 (3.8-4.0)
Female	Cutaneous abscess	197	46.8	2.0	100.2 (86.7-115.2)
	Cellulitis	88	20.9	1.2	76.3 (61.2-94.0)
	Phlebitis and thrombophlebitis	62	14.7	0.4	150.2 (115.2-192.6)
	Septicaemia and bacteraemia	18	4.3	0.6	30.4 (18.0-48.1)
	Osteomyelitis and septic arthritis	11	2.6	0.0	265.5 (132.5-475.0)
	Endocarditis	31	7.4	0.2	156.7 (106.5-222.4)
	Necrotising Fasciitis	**	**	**	1,460.1 (474.1-3,407.3)
	All bacterial infections*	405	96.3	4.4	92.8 (84.0-102.3)
	All-cause	3,179	755.7	893.9	3.6 (3.4-3.7)
Total	Cutaneous abscess	487	30.0	9.3	52.4 (47.8-57.2)
	Cellulitis	282	17.4	7.8	36.0 (31.9-40.4)
	Phlebitis and thrombophlebitis	233	14.3	2.0	115.2 (100.9-131.0)
	Septicaemia and bacteraemia	56	3.4	2.3	24.3 (18.3-31.5)
	Osteomyelitis and septic arthritis	42	2.6	0.2	174.4 (125.7-235.8)
	Endocarditis	82	5.0	1.9	43.7 (34.8-54.3)
	Necrotising Fasciitis	9	0.6	<0.1	599.4 (274.1-1,137.9)
	All bacterial infections*	1,180	72.7	23.6	50.0 (47.2-52.9)
	All-cause	9,274	571.0	2,467.5	3.8 (3.7-3.8)

* The total number of bacterial infections is less than the sum of each individual diagnosis because some admissions have two primary diagnoses (resulting from the process of merging hospital admissions that were within two days of each other)

** Suppressed to avoid disclosure of small counts

2 Comparison of matched hospital admissions

The cohort of people who inject heroin had a study entry point (their first episode of treatment) and a period of follow-up. We compared rates of hospital admission with rates in the general population, based on data for all hospital admissions among residents of local areas and general population estimates. Individuals in the comparison group therefore did not have a defined study entry point, meaning that the characteristics of hospital admissions could not be compared directly. We therefore sampled the same number of admissions from the study cohort and the general population within strata of age group, sex, year of admission and primary cause of admission (abscess, cellulitis, phlebitis, septicaemia, osteomyelitis, endocarditis, or necrotising fasciitis), and compared these admissions in terms of duration, method of admission, and method of discharge.

Table: Characteristics of hospital admissions for bacterial infections among people who inject drugs and the general population. Groups are matched on age group, sex, primary diagnosis and year of admission.

**Variable**	**Level**	**People who inject drugs n (%)**	**General population n (%)**
Total		1187 (100%)	1187 (100%)
Duration (days)	1	196 (17%)	440 (37%)
	2-4	455 (38%)	449 (38%)
	5-9	264 (22%)	179 (15%)
	10+	272 (23%)	119 (10%)
	Median (IQR)	4 (2-9)	2 (1-5)
	Mean (sd)	7.4 (9.9)	4.6 (8.1)
Admission method	Emergency	1145 (96%)	1024 (86%)
	Planned or other	42 (4%)	163 (14%)
Discharge method	On clinical advice	1020 (86%)	1168 (98%)
	Self-discharged	159 (13%)	11 (1%)
	Died	8 (1%)	8 (1%)

Figure: Duration of hospital admissions for treatment of bacterial infections. Hospital admissions are matched on age, sex, primary diagnosis and year of admission.

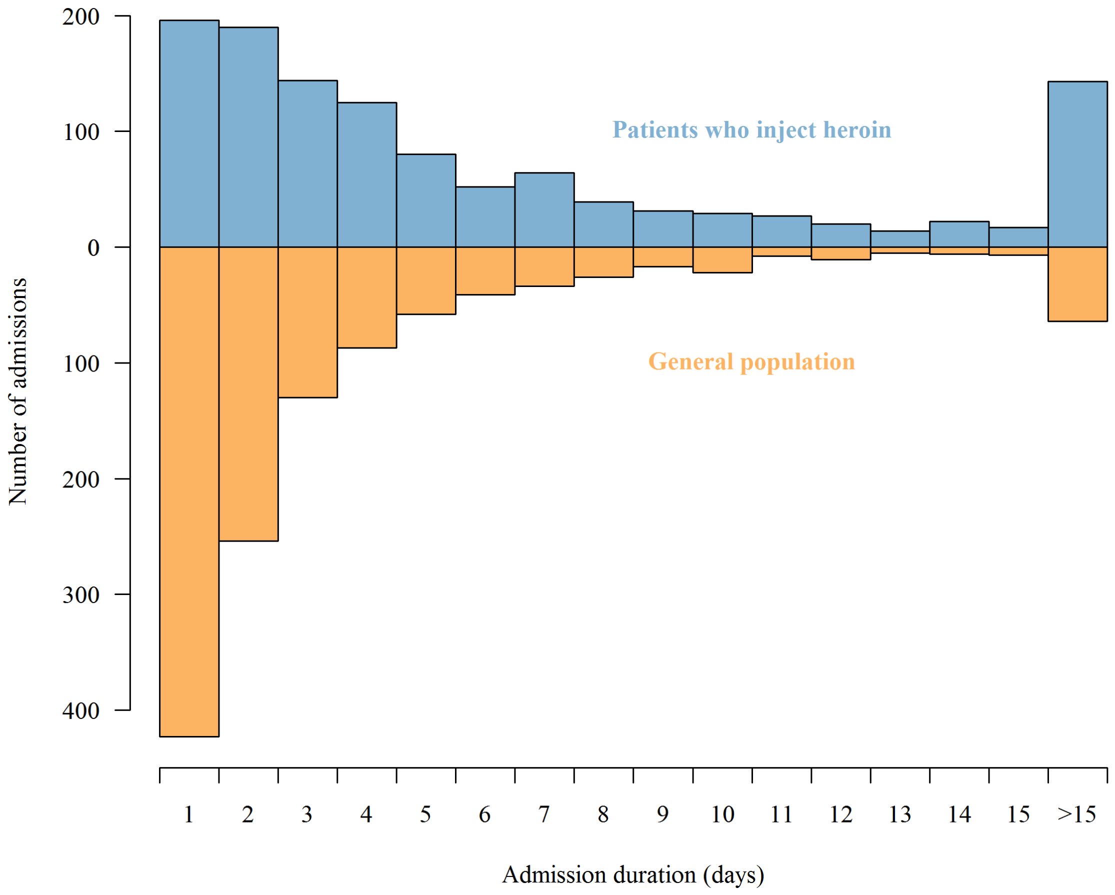

3 Modelled annual costs for London

Our data provides information on the number of admissions for severe bacterial infections for a cohort in South London over a period of about ten years. We wanted to estimate the number of admissions for the whole of London for one year.

We first took existing capture-recapture estimates of the number of people who inject drugs in London, 2011/12 (Hay et al., 2014), stratified by age group (15-24, 25-34 and 35-64). We then applied our estimates of the age-specific rates of admission for each primary diagnosis to estimate a number of admissions for London in one year. Finally, we applied the mean cost of admission for each primary diagnosis in our cohort (2014/15 prices).

We used a Monte-Carlo method to estimate uncertainty. We used the confidence intervals supplied with the capture-recapture estimates, sampling 10,000 values from a log-normal distribution to match the published confidence intervals as closely as possible. For the rates in our study cohort, we sampled 10,000 values of the number of admissions from a poisson distribution with a mean of the observed number, using the time-at-risk to calculate a rate in each simulation. For each simulation we calculated a cost, and then reported the 0.025 and 0.975 quantiles (the median value was very similar to the calculated value).

Table: modelled annual numbers of hospital admissions for bacterial infections among people who inject drugs, with costs, London, 2014/15

**Diagnosis**	**Estimated admissions**	**Mean admission cost**	**Total cost, £m (95% CI)**
Cutaneous abscess	347	4,304	1.50 (1.30-1.71)
Cellulitis	204	3,566	0.73 (0.61-0.85)
Phlebitis and thrombophlebitis	171	3,252	0.56 (0.46-0.67)
Septicaemia and bacteraemia	40	8,687	0.34 (0.24-0.46)
Osteomyelitis and septic arthritis	58	14,134	0.82 (0.16-1.53)
Endocarditis	35	12,963	0.45 (0.31-0.61)
Necrotising Fasciitis	7	10,815	0.08 (0.03-0.15)
Total	862	5,188	4.47 (3.68-5.34)
